# The role of cytochrome P450 genetic variants in pesticide metabolism and the risk of child neurodevelopment: a systematic review

**DOI:** 10.3389/fped.2025.1651916

**Published:** 2025-10-13

**Authors:** Peter M. Chilipweli, Benson R. Kidenya, Elias C. Nyanza, Aiwerasia Vera Ngowi, Karim Manji

**Affiliations:** ^1^ Department of Community Medicine and Environmental Health, School of Public Health, Catholic University of Health and Allied Sciences (CUHAS), Mwanza, Tanzania; ^2^ Department of Biochemistry and Molecular Biology, School of Medicine, Catholic University of Health and Allied Sciences (CUHAS), Mwanza, Tanzania; ^3^ Environmental and Occupational Health Department, School of Public Health, Muhimbili University of Health and Allied Sciences (MUHAS), Dar es Salaam, Tanzania; ^4^ Department of Pediatric and Child Health, School of Medicine, Muhimbili University of Health and Allied Sciences (MUHAS), Dar es Salaam, Tanzania

**Keywords:** neurodevelopment, cytochrome P450 variants, pesticide exposure, children, cognitive and motor development

## Abstract

**Introduction:**

Child neurodevelopment is influenced by genetic variants in cytochrome P450 (CYP450) enzymes, which affect detoxification and oxidative stress pathways. These variations modulate susceptibility to environmental toxins, influencing brain development through gene–environment interactions that impact neurogenesis, synaptogenesis, and inflammation, potentially contributing to neurodevelopmental disorders. This review has a major question to answer: how do CYP450 gene polymorphisms modulate pesticide metabolism and contribute to neurodevelopmental risks in children?

**Method:**

This study has been conducted according to PRISMA and includes studies conducted from 2005 to 2025. It has been registered to PROSPERO with the number PROSPERO 2024 CRD42024617478. The databases used for the search included PubMed/MEDLINE, Embase, Scopus, and African Journals Online (AJOL). Additional sources, such as OpenGrey, institutional repositories, and conference proceedings, were also searched using targeted search strategies and robust inclusion criteria, which were employed in study selection and final review to analyze gene–environment interactions and developmental outcomes.

**Results:**

A total of 758 studies were identified through database searches and manual reference checks. After eliminating studies due to various factors, 176 eligible studies were selected by reviewing the titles and abstracts. Finally, a total of nine articles were included in this systematic review. The review identified CYP3A4 as the most polymorphic gene, followed by CYP2C9, CYP2B6, and CYP1A2—all involved in pesticide metabolism, particularly organophosphates such as chlorpyrifos. Evidence from nine studies suggests that CYP450 polymorphisms influence pesticide detoxification capacity, increasing neurodevelopmental risks in exposed children. These findings highlight the importance of genetic risk assessment in public health strategies targeting pesticide-exposed populations.

**Conclusion:**

This review highlights the role of CYP450 gene polymorphisms in increasing susceptibility to pesticide-related neurotoxicity, especially in children and agricultural workers, emphasizing the need for genetically based risk assessment in public health strategies.

**Systematic Review Registration:**

PROSPERO CRD42024617478.

## 1 Introduction

Environmental factors play a crucial role in shaping neurodevelopment, with growing evidence suggesting that genetic predisposition alone cannot fully explain the increasing prevalence of neurodevelopmental disorders worldwide (1, 2). Exposure to pesticides, particularly during early childhood, has been strongly linked to adverse neurodevelopmental outcomes, especially in agricultural regions where children are frequently exposed through various exposure mechanisms such as maternal diet, transplacental transfer, breastfeeding, or inhalation of pesticide residues, contaminated water sources, or direct contact with agricultural environments (3). Unlike adults, children under 5 years are particularly vulnerable to environmental toxicants during critical periods of rapid brain growth and neurodevelopment due to their developing nervous systems, higher metabolic rates, and immature detoxification pathways (4, 5). The first 5 years are considered the most sensitive developmental window, where pesticide exposure and genetic susceptibility can exert lasting effects on cognitive, motor, and language outcomes. The extent to which pesticides impact neurodevelopment depends on various factors, including exposure duration, dosage, and an individual's ability to metabolize and eliminate these toxicants.

The biotransformation of pesticides in the human body is largely mediated by metabolic enzymes, with the cytochrome (CYP) P450 superfamily playing a central role in both the detoxification and bioactivation of these compounds. The cytochrome P450 (CYP450) enzymes are involved in Phase I metabolism, where they oxidize pesticides into intermediate metabolites, which are then further processed by Phase II enzymes for excretion (6). However, genetic polymorphisms in CYP genes, which are one of the xenobiotic metabolism enzymes (XME), can lead to significant variations in enzymatic activity, which in turn influence pesticide metabolism efficiency and neurotoxic risk. Some CYP variants result in poor metabolism, leading to the prolonged accumulation of toxic pesticide metabolites in the body, whereas others may enhance bioactivation, increasing the formation of neurotoxic intermediates (3). These genetic differences contribute to interindividual variability in pesticide-induced neurodevelopmental impairments.

Pesticides exert their neurotoxic effects through multiple mechanisms, disrupting key biological processes that are critical for normal brain development. One primary mechanism is acetylcholinesterase (AChE) inhibition (5, 7–9), which is particularly relevant for organophosphate pesticides. AChE is an essential enzyme responsible for breaking down acetylcholine, a neurotransmitter involved in synaptic communication, learning, and memory. When pesticides inhibit AChE, acetylcholine accumulates at synapses, leading to overstimulation of neural pathways, excitotoxicity, and neuronal cell death (4). This process has been linked to cognitive deficits, attention disorders, and neurobehavioral abnormalities in children exposed to organophosphates during critical periods of brain development. A study by Chen et al. (6) assessed the polymorphism effect on CYP3A4 and CYP2B6 metabolites, whereby the effect leads to drug metabolism and efficiency being compromised and a greater risk to neurodevelopment.

Another major mechanism is oxidative stress and mitochondrial dysfunction, where pesticide exposure leads to an overproduction of reactive oxygen species (ROS), disrupting mitochondrial function and inducing neuronal damage. Mitochondria, often referred to as the powerhouse of the cell, are essential for energy production and neuronal survival (6, 9–12). Excessive oxidative stress not only damages mitochondrial DNA but also triggers inflammation, apoptosis (programmed cell death), and impaired neural connectivity—all of which contribute to neurodevelopmental disorders such as autism spectrum disorder (ASD) and attention-deficit/hyperactivity disorder (ADHD) (3). In addition, pesticides act as endocrine disruptors, interfering with hormonal signaling pathways crucial for neurodevelopment. Thyroid hormones, for example, play a significant role in brain growth, synaptogenesis, and myelination. Pesticides that disrupt thyroid function may lead to altered neurodevelopmental trajectories, increasing the risk of cognitive impairments and behavioral disorders (13).

Furthermore, epigenetic modifications have emerged as an important factor in pesticide-induced neurotoxicity. Pesticides can alter gene expression by affecting DNA methylation patterns, histone modifications, and microRNA regulation. These epigenetic changes can have long-term consequences on brain function, potentially affecting multiple generations (13). Importantly, genetic variants in CYP genes may interact with these epigenetic modifications, influencing an individual's susceptibility to pesticide exposure and the severity of neurodevelopmental outcomes.

Given the central role of CYP enzymes in pesticide metabolism, CYP gene polymorphisms have been widely studied in the context of neurodevelopmental disorders. Several key CYP genes have been implicated in differential pesticide metabolism and neurodevelopmental risks. CYP2D6 polymorphisms, for instance, affect the enzyme's efficiency in metabolizing pesticides, with some variants leading to poor metabolism and increased retention of neurotoxic compounds. CYP1A2 enzyme, which is located in the liver and involved in caffeine N3-demethylation and the metabolism of polycyclic aromatic hydrocarbons and environmental contaminants, has polymorphic variants that may heighten neurodevelopmental vulnerability to pesticide exposure (3). CYP2E1, which plays a role in oxidative stress responses, is another important enzyme; its polymorphisms may contribute to higher ROS production, thereby exacerbating pesticide-induced neuronal damage. Similarly, CYP3A4/5 genes are crucial for metabolizing organophosphates and other xenobiotics, and their genetic variations can alter detoxification rates, thereby influencing the risk of neurodevelopmental impairments (6).

Children carrying specific CYP polymorphisms may exhibit significant variations in neurodevelopmental trajectories due to differences in pesticide biotransformation efficiency. Studies have shown that these CYP genetic variants influence the severity of neurodevelopmental impairments, including lower IQ scores, deficits in executive function, and an increased risk of neurodevelopmental disorders such as ASD and ADHD (6). In addition, maternal pesticide exposure during pregnancy, combined with certain CYP polymorphisms, may have transgenerational effects, where genetic susceptibility to pesticide toxicity is passed down, further increasing the risk of neurodevelopmental disorders in future generations.

Understanding the role of CYP450 polymorphisms in pesticide metabolism is essential for identifying at-risk populations and developing precision medicine approaches to mitigate neurodevelopmental risks among the population at risk. By recognizing genetic susceptibility factors, public health strategies can be tailored to protect vulnerable children from harmful pesticide exposure. Expanding knowledge in this field will be crucial for shaping evidence-based policies and public health interventions aimed at reducing neurodevelopmental harm from environmental toxins. This review specifically seeks to address the question of how CYP450 gene polymorphisms modulate pesticide metabolism and contribute to neurodevelopmental risks in children. Thus, the general objective of this review is to assess the influence of CYP450 genetic polymorphisms and pesticide exposure on neurodevelopmental outcomes in children under 5 years.

## 2 Methods

### 2.1 Search strategy for systematic review

This review study employs a standardized framework for systematic search and reporting strategy to review literature, which is the Preferred Reporting Items for Systematic Review and Meta-Analysis (PRISMA) (Supplementary Material S1). The study search was done based on PICO, whereby the population was children under 5 years, particularly those in agricultural settings or born to pesticide-exposed mothers. Intervention or exposure was prenatal and early-life exposure to pesticides, with metabolic processing influenced by CYP450 genetic variants, but the comparator was children with similar exposure but without the high-risk CYP450 variants or children with minimal pesticide exposure regardless of genotype. The outcome was neurodevelopmental outcomes.

A search was conducted to find studies on the role of cytochrome P450 genetic variants in pesticide metabolism and the risk of child neurodevelopment. Articles considered for the review were published between January 2005 and September 2025. Thus, the decision to include studies published from 2005 onward was made because of significant advancements in genomic and biomarker research methods in the early 2000s, which improved the accuracy of detecting CYP450 polymorphisms and pesticide exposure. The search was conducted across multiple databases, including PubMed/MEDLINE, Embase, Scopus, Web of Science, and African Journals Online (AJOL). Additional sources such as OpenGrey, institutional repositories, and conference proceedings were also explored.

The search utilized specific keywords related to CYP450, neurodevelopmental disorders, and the pediatric population. Boolean operators were applied to refine search queries, ensuring relevant studies were retrieved (Supplementary Material S3).

Neurodevelopment filters were set to include studies published in English from January 2005 to 2025, focusing on children under 5 years of age regardless of the country. The primary search query incorporated genetic markers, which were cytochrome P450, pesticides, neurodevelopmental disorders, and age-related filters to capture relevant studies.

A comprehensive gray literature search was conducted to complement published evidence on the role of cytochrome P450 genetic variants in pesticide metabolism and their potential impact on child neurodevelopment. Initially, preprints, non-peer-reviewed articles, studies with poor methodological rigor, and studies with a high risk of bias were excluded.

The search targeted global sources of gray literature, including institutional repositories of leading universities and research organizations across various regions. These included repositories as Harvard University, Johns Hopkins University, The London School of Hygiene & Tropical Medicine, and other global institutions with public research archives, the Open University of Tanzania (https://repository.out.ac.tz/), Catholic University of Health and Allied Sciences (CUHAS), Makerere University, Uganda, and University of Cape Town, South Africa.

Additional gray literature sources included official websites and technical report archives of international and national agencies such as the World Health Organization (WHO), Food and Agriculture Organization (FAO), UNICEF, and Ministries of Health, Agriculture, and Environment across different countries.

Abstract books and conference proceedings from major global scientific events were reviewed, including AFREhealth Conferences (https://afrehealth.org/), International AIDS Conferences, Global Neurodevelopmental and Environmental Health Symposia, and the International Society of Exposure Science (ISES). This global search approach ensured inclusion of unpublished theses, government reports, and conference abstracts with valuable insights into pesticide metabolism, genetic susceptibility, and child neurodevelopmental risks across different contexts and populations.

### 2.2 Review objectives and framework

The general objective for this review was to assess the influence of CYP450 genetic polymorphisms and pesticide exposure on neurodevelopmental outcomes in children under 5 years. But the specific objectives were to identify the CYP450 genetic variants most strongly associated with altered pesticide metabolism and increased neurotoxic effects in exposed children, to determine the combined effects of maternal pesticide exposure and CYP450 polymorphisms on prenatal and early childhood neurodevelopmental outcomes, and to examine the key gene environment interactions between CYP450 polymorphisms and pesticide exposure that contribute to cognitive and behavioral impairments in children.

The PICOS framework was utilized to structure the questions, defining the population, interventions/exposure, comparison, outcome, and study design as shown in Table 1 below. This review was registered in PROSPERO in 2024 with the number identification in PROSPERO 2024 CRD42024617478 (Supplementary Material S2).

**Table 1:** PICOS framework for the review.

Component	Description
Population (P)	Children under 5 years of age
Intervention/exposure (I)	Genetic variants of CYP450 and pesticide exposure as environmental factors
Comparison (C)	Children without CYP450 genetic variants or with minimal pesticide exposure
Outcomes (O)	Neurodevelopmental indicators, including cognitive, motor, and language development
Study design (S)	Observational studies (cross-sectional, cohort, case–control), randomized controlled trials (RCTs)

### 2.3 Study selection and eligibility criteria

Studies were included if they focused on children under 5 years old and pregnant women whose children were followed for neurodevelopmental outcomes. Research had to examine cytochrome P450 genetic variants and report on neurodevelopmental disorders such as cognitive delays, motor impairments, and language deficits. Environmental exposures of interest included pesticide exposure. Eligible study designs included observational (cohort, case–control, cross-sectional) and randomized controlled trials.

Studies were included based on the years in which they were published from 2005 to 2025 were selected for review, and the articles must be written in English.

To ensure methodological rigor, studies were excluded if they focused exclusively on the population with children under 5 years with other effects from exposure other than neurodevelopment, adults, or populations above 5 years; those that did not examine relevant genetic factors, lacked neurodevelopmental outcome assessments, were not published in English, or lacked methodological transparency; and editorials, commentaries, and reviews without primary data.

Thus, the literature search strategy was developed by PC, who is the first author of this paper, and was approved by a supervisor, EN, who is a co-author of this paper. Articles were screened for inclusion eligibility in this study by PMC and confirmed by EN. Two reviewers (PC and EN) reviewed the title, abstracts, and full-text for eligibility of the studies to be included in the current study, and a third reviewer checked for discrepancies (KM).

**Figure 1:**
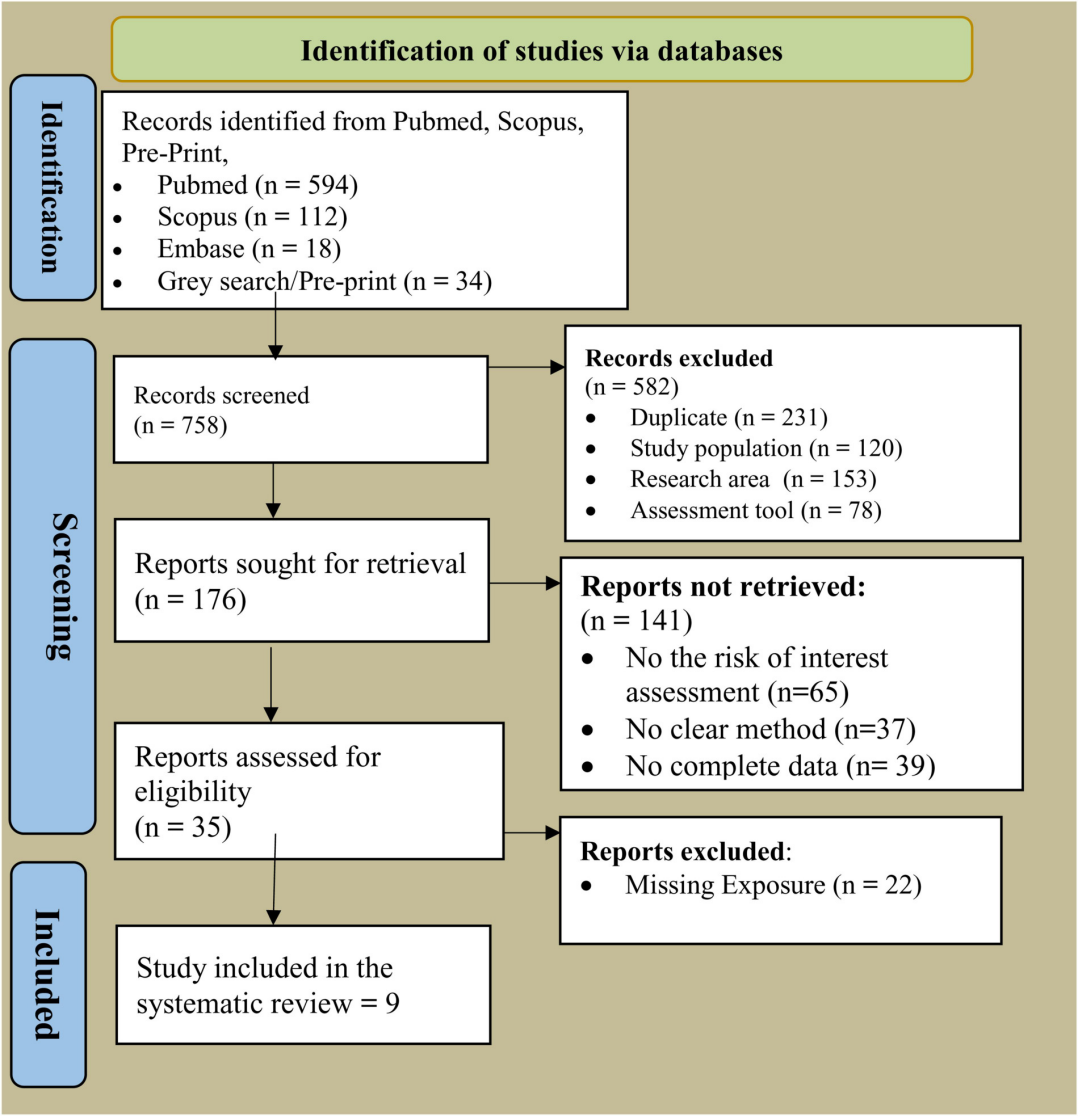
PRISMA diagram of the review publication selection process.

The online search identified 897 relevant citations. The titles and abstracts of 560 articles were reviewed in detail; 337 articles that did not meet the inclusion criteria were excluded from the review. After a thorough review of the titles and abstracts of articles, the full-texts of nine articles were retrieved and screened. Figure 1 summarizes the online search using the PRISMA flow diagram.

### 2.4 Data extraction and quality assessment

All the studies were uploaded to Covidence for data extraction and screening. A structured approach was used for study selection, data extraction, and quality assessment. Two independent reviewers screened studies in two stages—title/abstract review followed by full-text review. Discrepancies were resolved through consensus or consultation with a third reviewer, with the study selection process documented using a PRISMA flow diagram.

Data extraction focused on study characteristics (author, year, design, setting), population details (sample size, demographics), genetic factors (cytochrome P450 variants), environmental exposures (pesticides), neurodevelopmental outcomes (cognitive, motor, language assessments), and key findings (effect estimates, significance). A standardized extraction form was used to ensure consistency, and extracted data were stored in a secure database.

Data extraction and presentation. Data from the selected articles were extracted into an Excel spreadsheet. The Excel spreadsheet was structured to visualize the alignment of selected articles with the inclusion and exclusion criteria. PC performed the initial synthesis of collected data, and EN verified and checked the reported findings on the data. Data were extracted relating to the study title, authors, publication year, sample size, study design, biological sample analyzed, neurobehavioral tests used, age of children assessed, and outcomes of neurobehavioral tests (see Table 2). Data synthesis and analysis. Data from articles selected to be included were tabulated to represent study sample size, study design, screening tool used to measure pesticide of interest, genotype of interest, neurodevelopmental tool used, and the age at which it was administered. The abovementioned parameter was then compared among the articles selected for inclusion in the study.

**Table 2:** Summary of studies investigating CYP450 polymorphisms in pesticide metabolism and neurodevelopmental risk.

SN	Author	Year	Country	Study design	Pesticide type	CYP 450 alleles	Findings
1	Gómez-Martín et al. (20)	2015	Spain	Cross-sectional	Organophosphates	CYP2C19, CYP2D6, CYP3AP1	Adverse genotype combinations increase susceptibility to OP exposure
2	Sams et al. (21)	2000	UK	Experimental	Chlorpyrifos, parathion, diazinon	CYP3A4, CYP2D6	CYP3A4 and CYP2D6 play key roles in the bioactivation of organophosphates
3	Singh et al. (22)	2012	India	Case–control	Organophosphates	CYP2C9, GSTM1, GSTT1, NAT2	Certain genotypes (e.g., GSTM1 null, slow NAT2 acetylators) correlated with increased DNA damage in pesticide-exposed workers
4	Pedroni et al. (23)	2024	Italy	Computational	Chlorpyrifos	CYP2C19, CYP2B6	CYP2C19 plays a key role in detoxifying 6-chlorpyrifos, while CYP2B6 bioactivates it into toxic metabolites
5	Lind et al. (24)	2017	Sweden	Genome-Wide Association Study (GWAS)	Dichlorodiphenyltrichloroethane (DDT)/Dichlorodiphenyldichloroethylene (DDE)	CYP2B6	CYP2B6 variants significantly influence circulating DDE levels
6	Dadson et al. (25)	2013	USA/Egypt	Experimental	Profenofos	CYP3A4, CYP2B6, CYP2C19	CYP2C19 and CYP2B6 are primarily responsible for profenofos detoxification
7	Mutch et al. (26)	2003	UK	Experimental	Parathion	CYP3A4/5, CYP2C8, CYP1A2, CYP2D6	CYP3A4/5 and CYP2C8 are the primary enzymes that metabolize parathion to toxic paraoxon
8	Yang et al. (27)	2009	USA	Experimental	Pyrethroids	CYP3A4	Pyrethroids induce CYP3A4 expression and interact with pregnane X receptor (PXR), affecting metabolism and toxicity
9	Thistle et al. (4)	2022	Norwegian Mother, Father, and Child (MoBa)	Cohort study	Organophosphorus pesticides (OPPs)	CYP1A2 (1548T > C), CYP1A1 (IntG > A), CYP2A6 (-47A > C)	Higher prenatal OPP exposure is linked to poorer executive function in preschool children

### 2.5 Data synthesis and statistical analysis

Data synthesis involved a descriptive summary of study characteristics, categorized by exposure type (pesticides, exposures), genetic factors (cytochrome P450 polymorphisms), risk factors as the outcome measures (cognitive, motor, language development), and study design. The findings were compiled into a structured data table to facilitate comparative analysis. Since the studies used different methods and measures of effect, a meta-analysis was not conducted.

### 2.6 Quality appraisal

Quality assessment tools for bias were employed, including the risk of bias in non-randomized studies (ROBINS V2), which helps identify how bias could distort results in non-randomized studies. This was used to evaluate how CYP450 genetic variants modify pesticide effects, the interaction between genes and environment, and the accuracy and validity of exposure and outcome assessment. The Critical Appraisal Skills Programme (CASP) Checklist, which is the most widely used tool for assessing qualitative research, was used to appraise the quality of articles for inclusion in the review. These guidelines assess the quality of studies based on selection bias, study design, confounders, blinding, data collection tools, withdrawal and dropouts, intervention integrity, and analysis.

The first author (PC) performed the quality appraisal for articles that were in line with our current study; then, the reviewer (EN) performed the quality appraisal according to his judgment for the selected articles. The reviewers then cross-checked if the appraisal had any differences, which were settled by the third reviewer (BK) (as he is most experienced in this study).

## 3 Results

### 3.1 Study selection

A total of 758 studies were identified through database searches and manual reference checks. After eliminating studies due to various factors, 176 eligible studies were selected by reviewing the titles and abstracts (Figure 1). Finally, a total of nine articles were included in this systematic review (Figure 1).

### 3.2 Type of CYP450

**Figure 2:**
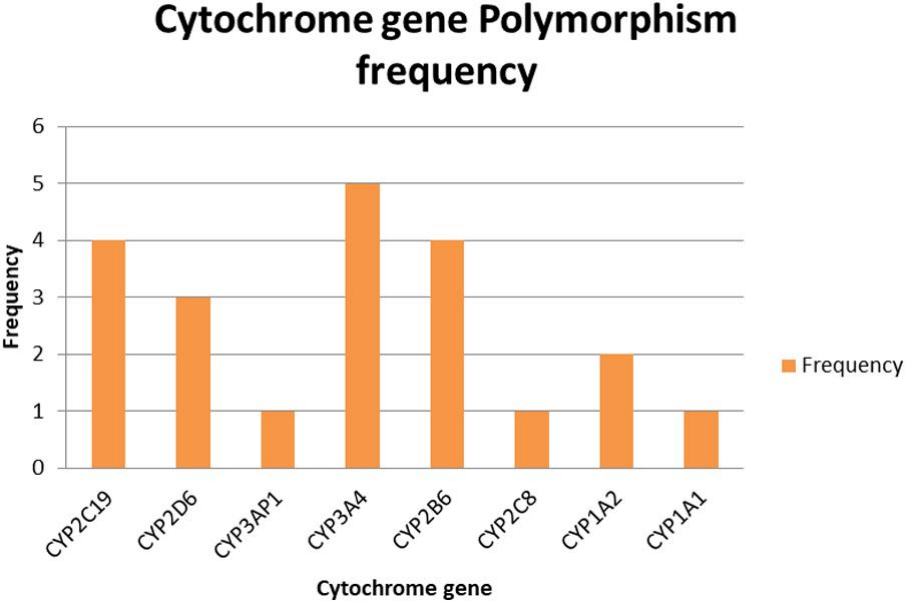
Types of gene alleles of concern.

Among the cytochrome P450 genes analyzed from the paper, CYP3A4 exhibited the highest level of polymorphism, indicating it as the most variable gene within the study population. This gene is well known for broad substrate specificity, metabolizing over 50% of clinically used drugs and various pesticides. Both CYP2C9 and CYP2B6 were observed with a polymorphism frequency of 4, suggesting a moderate degree of variability. However, CYP2B6 plays a key role in the metabolism of specific organophosphates, such as chlorpyrifos (Figure 2). In addition, CYP1A2 displayed an intermediate polymorphism. This gene is essential for the metabolism of polycyclic hydrocarbons and is inducible by environmental exposures such as pesticides (Figure 2).

### 3.3 Pesticides, cytochrome P450, and the risk of neurodevelopmental disorder

A total of nine studies were included in the review, whereby most of them had high diversity in their methodology and measure of effect which makes them not suitable for meta-analysis. However, studies have shown that most of the pesticides that participants were exposed to include the organophosphates such as profenofos, parathion, diazinon, and chlorpyrifos, which are AChE inhibitors.

This review highlights the critical role of CYP450 gene polymorphisms in modulating the metabolism and toxicity of pesticides, particularly organophosphates, and their potential contribution to neurodevelopmental risks in children. Across the included studies, several CYP450 enzymes—such as CYP2C19, CYP2D6, CYP3A4, CYP2B6, and CYP1A2—emerged as central players in either the detoxification or bioactivation of pesticides (Table 2).

Polymorphisms in these genes were consistently associated with altered metabolic capacity, leading to variations in internal pesticide dose, accumulation of toxic metabolites, and, in some cases, genotoxic damage. Notably, adverse genotypes (e.g., GSTM1 null or slow NAT2 acetylators) compounded the risks, especially in vulnerable populations such as agricultural workers and exposed children.

Moreover, prenatal exposure to organophosphates in conjunction with specific CYP450 variants was linked to impaired executive function in early childhood, underscoring the intersection between genetic susceptibility and environmental exposure in shaping neurodevelopmental outcomes. Thus, these findings emphasize the need to identify genetically at-risk populations and incorporate genetic screening and risk stratification into public health strategies, particularly in regions with high pesticide use and limited regulatory oversight.

## 4 Discussion

Pesticide exposure, particularly occupational and environmental exposure to organophosphates, is widely recognized as a major public health concern due to its association with a broad spectrum of health effects, especially among vulnerable populations such as children (13). Understanding individual susceptibility to these exposures is critical for designing targeted interventions. One promising approach is the evaluation of polymorphisms in genes encoding for enzymes responsible for pesticide metabolism, notably the CYP450 family.

Under this consideration, several studies have pointed out that organophosphates such as profenofos, parathion, diazinon, and chlorpyrifos were the predominant pesticides reported among exposed participants. These compounds exert their toxicological effects primarily through the inhibition of AChE, leading to neurotoxicity (9). Phosphorothioate OPs are metabolized by cytochrome P450s (P450s) through either a dearylation and desulfuration reaction to form an inactive metabolite, or an active oxon metabolite, which is a potent cholinesterase inhibitor (14). Both reactions are facilitated by CYP450; this point is significant in detoxification and metabolizing pesticides in the body.

In this study review, CYP3A4 was identified as the gene with the highest level of polymorphism among participants, suggesting that it is the most variable gene in the population under investigation. This finding is consistent with previous studies, such as that by Zhang et al. (15), which highlighted that the CYP3A4*22 variant could significantly influence drug and pesticide metabolism. Polymorphisms in CYP3A4 are known to result in reduced enzymatic activity, subsequently impairing the breakdown and clearance of pesticides from the body (5, 9, 16). Such impaired metabolism could lead to greater bioaccumulation of toxicants and heighten the risk of neurodevelopmental risks to vulnerable populations such as children.

Moreover, CYP2B6 was emphasized as playing a key role in the biotransformation of specific organophosphates, such as chlorpyrifos, an AChE inhibitor widely used in agriculture (17, 18). This enzyme's variability may therefore critically determine interindividual differences in pesticide detoxification and susceptibility to toxicity. Similarly, polymorphisms in CYP2D6 have been implicated in pesticide metabolism and linked to increased risk for Parkinson's disease and pesticide-induced neurotoxicity among occupationally exposed populations. The findings from other studies (5, 9, 16) demonstrated that the CYP2D6 G1934A (rs3892097) variant was more frequent among rice farmers with long-term pesticide exposure and associated with lower serum cholinesterase levels—an established biomarker of organophosphate toxicity (9). As most of the studies pointed out that dearylation reaction facilitated by CYP450 is mostly done to organophosphate (parathion, chlorpyrifos, and diazinon); thus, it is important to consider CYP3A4 and CYP2B6 with the risk of neurodevelopment.

Polymorphisms in CYP1A1, CYP1A2, and CYP2A6 have also been studied in relation to organophosphate exposure and were associated with executive function deficits in children. For instance, Thistle et al. (19) observed that higher prenatal organophosphate exposures correlated with poorer executive function scores, particularly in emotional control and inhibition, in children carrying specific alleles of these genes. The importance of genetic variability was further illustrated by Docea et al. (3), who highlighted that polymorphisms in several CYP450 genes (CYP1A1, CYP1A2, CYP2C9, CYP2C19, CYP2D6, CYP3A4) could modulate susceptibility to neurotoxicity, endocrine disruption, immune dysfunction, and reproductive impairments following xenobiotic exposure. Moreover, Thistle et al. added the other aspect of consideration to CYP1A1, CYP1A2, and CYP2D6 as important alleles to consider in the metabolism of pesticides.

Collectively, these findings point toward a complex interplay between genetic susceptibility and environmental exposures, where polymorphisms in CYP450 genes modulate the metabolism, detoxification, and, consequently, the health outcomes following pesticide exposure. Notably, adverse genotypes, such as GSTM1 null or slow NAT2 acetylators, may compound the risk, particularly among agricultural workers and children exposed during critical periods of neurodevelopment.

The convergence of evidence across multiple studies emphasizes the pressing need for genetic screening strategies to identify at-risk populations. Incorporating genetic risk stratification into public health programs could enhance prevention strategies, particularly in settings characterized by heavy pesticide use and weak regulatory frameworks. Early identification of genetically susceptible individuals could inform tailored interventions, such as enhanced exposure protections, targeted monitoring, and personalized therapeutic approaches to mitigate adverse health effects. Moreover, these insights align with the growing emphasis on precision public health—leveraging genetic and environmental data to better predict, prevent, and manage health risks in populations.

### 4.1 Strength and limitations

#### 4.1.1 Strength of the study

• The review integrates toxicology, genetics, environmental health, and neurodevelopment, offering a holistic understanding of how CYP450 gene variants influence pesticide metabolism and health outcomes.
• Special attention to children and agricultural workers enhances the relevance of findings to public health and supports prioritization of vulnerable groups. Thus, this topic is timely and highly relevant, especially for regions with high pesticide usage and limited genetic monitoring systems.
• The review proposes actionable insights such as genetic screening, personalized interventions, and integration into public health policy, supporting translational application.
• Mechanistic pathways are well explained, particularly how CYP450 enzymes modulate pesticide activation/inactivation and subsequent neurotoxicity.

#### 4.1.2 Limitations of the study

• Many genetic polymorphism studies originate from non-African populations. The absence of large-scale, population-specific data from Africa may limit generalizability.
• The review may primarily include studies reporting significant associations, while null or negative findings may be underreported.
• Many included studies may not adequately control for confounding factors such as nutrition, co-exposures (e.g., heavy metals), socioeconomic status, or ART exposure in HEU children.
• Few studies comprehensively evaluate gene–environment interactions in the context of exposures to pesticides, which may limit full understanding of real-world risks.
• Inconsistent methods for measuring pesticide exposure (biomarkers, questionnaires, environmental sampling) across studies could introduce exposure misclassification.
• There is a scarcity of long-term follow-up data linking early-life exposure and genetic susceptibility with later neurodevelopmental outcomes.

## 5 Conclusion and recommendation

### 5.1 Conclusion

This review underscores the critical role of CYP450 gene polymorphisms in modulating individual susceptibility to pesticide toxicity, particularly for organophosphate compounds. Polymorphisms in genes such as CYP3A4, CYP2B6, CYP2D6, CYP1A2, and CYP1A1 significantly alter the metabolism and detoxification of pesticides, leading to variations in internal exposure levels, bioactivation of toxic metabolites, and subsequent health effects, including neurodevelopmental impairments.

Importantly, the evidence highlights that individuals with genotypes, such as CYP3A4*22 carriers or CYP2D6 G1934A variants, may experience diminished enzymatic activity, increasing their vulnerability to pesticide-related neurotoxicity and other adverse outcomes. The findings particularly raise concern for children, whose developing nervous systems are highly sensitive to environmental toxicants, and agricultural workers who experience chronic occupational exposures.

The intersection of genetic susceptibility and environmental pesticide exposure presents a compelling case for integrating genetic screening and personalized risk assessments into public health strategies, particularly in high-exposure settings with limited pesticide regulation and enforcement.

### 5.2 Recommendation

The health authorities should consider introducing targeted genetic screening for populations at high risk of pesticide exposure (e.g., agricultural workers, residents in farming communities) to identify individuals carrying high-risk CYP450 polymorphisms. Moreover, the Ministry of Health should strengthen occupational health surveillance by implementing regular monitoring of cholinesterase levels and pesticide exposure biomarkers for workers handling organophosphate pesticides, with enhanced follow-up for genetically susceptible individuals.

Interventions on promoting the use of safer alternatives to organophosphates, appropriate personal protective equipment (PPE), and integrated pest management (IPM) strategies should be intensified, especially in rural agricultural regions. The governments should strengthen pesticide regulations, ensuring registration, usage, and monitoring processes incorporate evidence on genetic susceptibility and cumulative exposure risks.

Public health policies should integrate precision public health approaches, considering genetic, environmental, and occupational factors to better predict and prevent adverse health outcomes associated with pesticide exposure.

Lastly, large longitudinal studies are recommended to better elucidate the gene–environment interactions between pesticide exposures and CYP450 polymorphisms, with a focus on neurodevelopmental outcomes in children and chronic diseases in adults. To conduct awareness programs targeting farmers and rural populations to educate them about the risks of pesticide exposure, genetic susceptibility, and preventive measures to reduce health impacts.

## Data Availability

The original contributions presented in the study are included in the article/Supplementary Material; further inquiries can be directed to the corresponding author.
